# Treatment Strategy and Residual Disease as Determinants of Survival in Stage IVB High‐Grade Serous Ovarian Cancer: A Retrospective Cohort Study

**DOI:** 10.1002/jso.70142

**Published:** 2025-11-24

**Authors:** Anouk Benseler, Adina Tanen, Taymaa May, Lisa Avery, Genevieve Bouchard‐Fortier, Marcus Q. Bernardini, Liat Hogen

**Affiliations:** ^1^ Department of Obstetrics and Gynecology University of Toronto Toronto Ontario Canada; ^2^ Temerty Faculty of Medicine University of Toronto Toronto Ontario Canada; ^3^ Department of Obstetrics and Gynecology, Harvard Medical School, Division of Gynecologic Oncology, Brigham and Women's Hospital Mass General Brigham Boston Massachusetts USA; ^4^ Department of Surgical Oncology, Division of Gynecologic Oncology Dana Farber Cancer Institute Boston Massachusetts USA; ^5^ Department of Biostatistics University Health Network Toronto Ontario Canada; ^6^ Dalla Lana School of Public Health, Biostatistics Division University of Toronto Toronto Ontario Canada; ^7^ Division of Gynecology Oncology University Health Network and Sinai Health System Toronto Ontario Canada

**Keywords:** cytoreductive surgery, high‐grade serous ovarian cancer, Stage IVB, survival

## Abstract

**Background and Objective:**

Stage IVB high‐grade serous ovarian cancer (HGSOC) carries a poor prognosis. We aimed to: (1) describe the characteristics and survival of patients treated with primary cytoreductive surgery (PCS), interval cytoreductive surgery (ICS) or chemotherapy alone, (2) investigate the correlation between disease distribution and treatment type, and (3) evaluate the impact of cytoreductive surgery (CS) “aggressiveness” and outcome on survival.

**Methods:**

A single‐center retrospective cohort study of Stage IVB HGSOC patients. Demographics, tumor characteristics, treatment including “aggressive” CS (upper abdominal and extraperitoneal procedures), and outcomes were analyzed using descriptive statistics and survival analysis, with nonparametric tests and Cox‐proportional hazard models.

**Results:**

Of 110 patients, 24 (22%) underwent PCS, 73 (66%) ICS, and 13 (12%) chemotherapy alone. Median overall survival (OS) was 76.2 (PCS), 36.9 (ICS), and 20.1 months (chemotherapy alone) (*p* = 0.014). Supradiaphragmatic lymph‐node metastasis differed across groups (*p* = 0.042). “Aggressive” CS was performed in 53.6% of the surgical cohort, with 54.86% no‐gross‐residual (NGR), 34% optimal ≤ 1 cm ≤ and 11.3% suboptimal/aborted surgical outcome. Median OS post CS for NGR, optimal ≤ 1 cm, and suboptimal was 67.55, 35.26, and 20.97 months, respectively (*p* = 0.006).

**Conclusions:**

OS for Stage IVB HGSOC follows a hierarchical pattern: PCS, ICS, and chemotherapy. Disease distribution guides treatment and residual tumor after CS correlates with survival.

## 1 Introduction

Historically, 12%–28% of women with ovarian cancer present with Stage IV disease, with a 5‐year survival of approximately 11%–18% [1, 2]. Over the past decade, the International Federation of Gynecology and Obstetrics staging for ovarian cancer has been refined to encompass peritoneal and fallopian tube cancers, while subdividing Stage IV into IVA (pleural effusion with positive cytology) and IVB (parenchymal metastases or extra‐abdominal metastases), including inguinal lymph node involvement [3]. Given these updated definitions, incidence and prognosis of Stage IVB high‐grade serous ovarian cancer (HGSOC) specifically remain undercharacterized in the literature [1, 3, 4, 5]. Published median overall survival (OS) for Stage IV ranges from 21 to 34 months, yet survival data focused exclusively on Stage IVB are scarce [6].

Standard therapy for advanced ovarian cancer typically involves a combination of cytoreductive surgery (CS) and platinum‐based chemotherapy [3]. The decision to pursue either primary cytoreductive surgery (PCS) or neoadjuvant chemotherapy followed by interval cytoreductive surgery (ICS) is mainly based on stage, disease extent, and patient performance status, with the principal surgical objective being to achieve no gross residual (NGR) disease [7, 8]. In Stage IVB disease, achieving NGR can be particularly challenging owing to metastatic disease in anatomical areas not easily resectable, extensive tumor burden, and/or poor patient performance status [9]. Moreover, whether the prognostic benefit of NGR observed in Stage III disease similarly exists in Stage IVB is uncertain, especially when thoracic or parenchymal liver resections are required to attain complete cytoreduction [10]. Additionally, prognosis is also influenced by tumor biology, histology, molecular and genetic markers, and comorbidities, adding further heterogeneity [11, 12].

Given these considerations, we designed a retrospective study to: (1) describe the clinical characteristics and survival outcomes of patients with Stage IVB HGSOC treated with primary surgery, neoadjuvant chemotherapy and interval surgery, or chemotherapy alone; (2) investigate the correlation between disease distribution and treatment choice; and (3) examine the impact of residual disease and surgical “aggressiveness” during cytoreductive surgery on survival outcomes.

## 2 Materials and Methods

This is a retrospective cohort study of women with newly diagnosed Stage IVB HGSOC at an academic tertiary care center. Patients treated between January 1, 2015 and July 1, 2020 were identified through an institutional database. Patients with Stage IVB HGSOC on biopsy or surgical specimen, or with qualifying metastasis of at least 1 cm on imaging prior to initiation of treatment, were included. Patients were excluded if they had Stage III or IVA disease, follow‐up < 3 months, or insufficient documentation of clinical data. This study was approved by the University Health Network Research Ethics Board (CAPCR ID 20‐5945.3).

### 2.1 Demographic, Tumor, and Functional Characteristics

Patient demographic information, tumor histology, *BRCA* mutation status, CA‐125 at diagnosis, Eastern Cooperative Oncology Group (ECOG) functional assessment score at diagnosis, and Stage IVB qualifying metastases were recorded. Supradiaphragmatic lymph node metastasis was defined as epiphrenic, cardiophrenic, pericardial, mediastinal, paratracheal, axillary, precarinal, hilar, retrocrural, internal mammary, paraesophageal, and/or supraclavicular lymph node involvement.

### 2.2 Oncologic Treatment Strategy Selection

Patients were categorized by initial treatment strategy: primary surgery, neoadjuvant chemotherapy followed by interval surgery (interval‐surgery group), or chemotherapy alone. For patients treated between January 2015 and October 2018, the choice of initial treatment was based on physician preference. From October 2018 onward, an ovarian multidisciplinary cancer conference guided treatment decisions. Disease was deemed unresectable if resection to no residual disease or residual disease ≤ 1 cm in diameter was not feasible, either by multidisciplinary review of preoperative imaging or by intraoperative assessment by a gynecologic oncologist.

### 2.3 Oncologic Treatment Characteristics

We documented the number of chemotherapy cycles, the use of bevacizumab and/or PARP inhibitors, and the surgical procedures performed at the time of primary surgery or interval surgery. Consistent with institutional practice, bevacizumab was added to the chemotherapy regimen only after interval surgery or if interval surgery was ultimately not performed. Surgeon‐assessed residual tumor size was recorded for primary surgery or interval surgery. An “aborted procedure” was defined as any surgery discontinued before maximal surgical effort. Women whose primary surgery was aborted and subsequently underwent neoadjuvant chemotherapy with interval surgery were analyzed in the interval‐surgery group, and those with an aborted interval surgery were also assigned to the interval‐surgery group. An “aggressive” CS included resection of one or more of the following: diaphragm, liver, spleen, thorax, neck, bowel (excluding appendectomy), para‐aortic lymph nodes, inguinal lymph nodes, or abdominal wall. Among patients managed with chemotherapy alone, posttreatment CA‐125 levels and the clinical reasons for not proceeding to interval surgery were collected.

### 2.4 Outcomes

Key study endpoints included OS and progression‐free survival. For each patient, these were calculated from the date of initial consult to the date of recurrence, the date of last follow‐up, or the date of death as applicable. Progression was defined radiologically based on RECIST 1.1 criteria using computed tomography scans [13] and confirmed pathologically as indicated based on clinician discretion.

### 2.5 Statistical Analysis

Descriptive statistics were used to summarize patient demographic, tumor, and functional score data, as well as chemotherapy and surgical details. Continuous variables were reported as medians with interquartile ranges (IQR) and range; categorical variables were presented as counts and proportions. OS was defined as the interval from initial consult to death from any cause or last follow‐up. Progression‐free survival was defined as the interval from initial consult to disease progression or death, whichever occurred first, and was censored at the last follow‐up for patients without progression or death.

Categorical comparisons of disease distribution were performed using Fisher's exact test, and the Kruskal–Wallis test was used for continuous variables. Kaplan–Meier curves were generated to visualize OS and progression‐free‐survival for the entire cohort and by treatment approach. Within the primary‐surgery and interval‐surgery cohorts, OS and progression‐free survival were further examined by residual disease outcome (NGR vs. optimal cytoreduction (≤ 1 cm) vs. suboptimal cytoreduction (> 1 cm)) and by procedure details in the interval‐surgery subset. The log‐rank test was applied for between‐group comparisons.

A Cox proportional hazards model with screening variable selection was fitted to compare OS and progression‐free survival between patients treated with interval surgery versus those receiving chemotherapy alone. Covariates with insufficient data for stable estimates were excluded. The proportional hazards assumption was tested for each model. Statistical significance was set at *α* = 0.05. All analyses were performed using R version 4.4.1 (R Core Team, Vienna, Austria).

In accordance with the journal's guidelines, we will provide our data for independent analysis by a selected team by the Editorial Team for the purposes of additional data analysis or for the reproducibility of this study in other centers if such is requested.

## 3 Results

### 3.1 Demographic, Tumor, and Functional Characteristics

A total of 110 patients with Stage IVB HGSOC were treated at our tertiary cancer care center between January 1, 2015 and July 1, 2020, and met the inclusion criteria. The median age was 63 (IQR 56–71) and 86% had an ECOG score of 0 or 1. Of these, 24 (22%) were treated with primary surgery, 73 (66%) were treated with NACT and interval surgery, and 13 (12%) were treated with chemotherapy alone. For patients who received neoadjuvant chemotherapy, each received a median of four cycles (range 1–6) (Table 1). The median (IQR) follow‐up time was 33.8 (21.2–58.7) months, ranging from 2.3 to 109.6 months. Age, ECOG scores, CA‐125, and *BRCA* status were not statistically significantly different between the three treatment groups, and specifically not statistically significantly different between the patients who received interval surgery versus chemotherapy alone.

**Table 1:** Demographic characteristics of patients with Stage IVB high‐grade serous ovarian cancer.

Characteristics	Total (*n* = 110)	PCS (*n* = 24)	NACT & ICS (*n* = 73)	Chemotherapy alone (*n* = 13)
Age, median (IQR)	63 (56–71)	61 (55–70)	64 (57–70)	62 (50–73)
ECOG score^a^				
0	57 (52%)	16 (67%)	34 (47%)	7 (54%)
1	37 (34%)	7 (29%)	26 (36%)	4 (31%)
2	12 (11%)	1 (4%)	10 (14%)	1 (8%)
3	4 (4%)	0	3 (4%)	1 (8%)
BRCA status (somatic or germline mutation)^a^, ^b^	20 (18%)	6 (26%)	12 (16%)	2 (15%)
CA125, median (IQR)^b^	817 (194–1610)	782 (171–1908)	811 (189–1559)	1345 (658–3909)
Neoadjuvant chemotherapy cycles, median (IQR)^b^	4 (4–6)	—	4 (3–6)	4 (4–5)
Bevacizumab^a^	48 (44%)	—	43 (59%)	5 (38%)

*Note:* The number of chemotherapy cycles was unknown for three patients treated with NACT and ICS.

Abbreviations: BRCA, breast cancer gene; ECOG, Eastern Cooperative Oncology Group; ICS, interval cytoreductive surgery; IQR, interquartile range; NACT, neoadjuvant chemotherapy; PCS, primary cytoreductive surgery.

^a^ Categorical data described by the number of participants (%).

^b^ BRCA status and serum CA125 were missing for one patient treated with PCS.

Eighty patients had only a single site of metastatic disease qualifying their distribution as Stage IVB. There were 10 patients with only liver parenchyma metastasis, 5 patients had only lung metastasis, 5 patients had only splenic parenchyma metastasis, 1 patient had only bone metastasis, 7 patients had only inguinal lymph nodes metastasis, and 52 patients had only supradiaphragmatic lymph node metastasis. The remaining 30 of the 110 patients had multiple metastatic sites of disease to qualify as Stage IVB.

Only the incidence of supradiaphragmatic lymph node metastasis differed between treatment groups (*p* = 0.042) (Table 2).

**Table 2:** Stage IVB disease distribution at time of diagnosis in patients with high‐grade serous ovarian cancer.

Disease sites qualifying for stage IVB at the time of diagnosis	Total (*n* = 110)	PCS (*n* = 24)	NACT & ICS (*n* = 73)	Chemotherapy alone (*n* = 13)	*p*
Parenchymal metastasis					0.19
Solitary ≥ 1 cm liver parenchyma lesion	12 (11%)	2 (8%)	9 (12%)	1 (8%)	
Multiple ≥ 1 cm liver parenchymal lesions	7 (6%)	1 (4%)	3 (4%)	3 (23%)	
Splenic lesion ≥ 1 cm	7 (6%)	3 (12%)	4 (5%)	0	0.40
Metastasis to extra‐abdominal organs					
Brain lesion	1 (1%)	0	1 (1%)	0	1.00
Lung parenchymal lesion					0.53
Single ≥ 1 cm lesion	4 (4%)	0	3 (4%)	1 (8%)	
Multiple ≥ 1 cm lesion	10 (9%)	2 (8%)	6 (8%)	2 (15%)	
Splenic lesion ≥ 1 cm	7 (6%)	3 (12%)	4 (5%)	0	0.40
Bone lesion ≥ 1 cm	5 (5%)	1 (4%)	2 (3%)	2 (15%)	0.13
LN metastasis outside the abdominal cavity					
Supradiaphragmatic LN ≥ 1cm					0.042
Cardiophrenic or epiphrenic LN	24 (22%)	5 (21%)	17 (23%)	2 (15%)	
Other supradiaphragmatic LN^a^	34 (31%)	7 (29%)	25 (34%)	2 (15%)	
Both cardiophrenic or epiphrenic LN, and other supradiaphragmatic LN	16 (15%)	0	11 (15%)	5 (38%)	
Inguinal LN ≥ 1cm	21 (19%)	3 (12%)	17 (23%)	1 (8%)	0.35

Abbreviations: ICS, interval cytoreductive surgery; LN, lymph node; NACT, neoadjuvant chemotherapy; PCS, primary cytoreductive surgery.

^a^ Other supradiaphragmatic LN included pericardial, mediastinal, paratracheal, axillary, precarinal, hilar, retrocrural, internal mammary, paraesophageal, and supraclavicular lymph nodes.

### 3.2 Oncologic Treatment Characteristics

A total of 97 surgical procedures were performed and had reported surgical outcome. Surgeons reported NGR surgical outcome in 53/97 (54.6%) (14/26 (53.9%) in primary surgery and 39/71 (54.9%) in interval surgery), optimal ≤ 1 cm in 33/97 (34%) (7/26 (26.9%) in primary surgery and 26/71 (36.6%) in interval surgery), and suboptimal/aborted in 11/97 (11.3%) (5/26 (19.2%) in primary surgery and 6/71 (8.5%) in interval surgery) (Table 3). Of the two patients who had aborted primary surgery, one patient had interval surgery after five cycles of neoadjuvant chemotherapy with optimal cytoreduction, and the second patient had chemotherapy only. Including patients that had chemotherapy only (*n* = 13), patients whose interval‐surgery procedure was aborted (*n* = 2) and patients who had suboptimal interval surgery (*n* = 4), a total of 19 patients (17%) in our cohort were poor surgical candidates.

**Table 3:** Residual tumor after cytoreductive surgery for patients with Stage IVB high‑grade serous ovarian cancer.

Surgical outcome	Primary cytoreductive surgery (*n* = 26)^a^	Interval cytoreductive surgery (*n* = 71)^b^	Total (*n* = 97)
No gross residual tumor	14 (53.9%)	39 (54.9%)	53 (54.6%)
Optimal cytoreduction (≤ 1 cm residual tumor)	7 (26.9%)	26 (36.6%)	33 (34%)
Suboptimal cytoreduction (> 1 cm residual tumor)/aborted procedures^c^	5 (19.2%)	6 (8.5%)	11 (11.4%)
Total	26	71	97

^a^ Two patients whose primary cytoreductive surgery was aborted went on to receive neoadjuvant chemotherapy and attempted interval cytoreduction; therefore, they were included in both the PCS and ICS groups in this table.

^b^ Cytoreductive surgical outcome was unknown for two patients who underwent interval cytoreductive surgery and they were excluded from this table.

^c^ Suboptimal/aborted procedures for the primary cytoreductive surgery group included two palliative procedures, two open‑and‑close procedures, and one suboptimal cytoreduction (> 1 cm residual). Suboptimal/aborted procedures in the interval cytoreductive surgery group included four cases with gross residual disease > 1 cm and two open‑and‑close procedures.

### 3.3 Outcomes

The median OS for the entire cohort was 42.4 months (95% CI: 34.1–67.5). Stratified by treatment group, median OS was 76.2 months (95% CI: 58.8–not estimable (NE)) for primary surgery, 36.9 months (95% CI: 32.6–57.2) for interval surgery, and 20.1 months (95% CI: 10.4–NE) for chemotherapy alone (*p* = 0.014). For the entire surgical cohort, (primary surgery and interval surgery), patients with NGR had median OS of 67.55 months (95% CI: 45.07, NE), patients with optimal ≤ 1cm 35.26 months (95% CI: 28.68, NE), and patients with suboptimal > 1 cm 20.97 months (95% CI: 7.4, NE) (*p* = 0.006).

In further analysis of OS by treatment group and residual disease, primary surgery with NGR had a median OS of 76.23 months (95% CI: 58.77, NE) and primary‐surgery optimal ≤ 1 cm NE (95% CI: 38.16, NE) (*p* = 0.36). Patients undergoing interval surgery with NGR had a median OS of 41.07 months (95% CI: 28.97, NE), and interval‐surgery optimal ≤ 1 cm ≤ 34.06 months (95% CI: 27.77, NE) (*p* = 0.211).

The median OS was similar between the *n* = 6 patients that had aborted/suboptimal cytoreduction at interval surgery (18.5 months, 95% CI: 7.4, NE) and the *n* = 13 patients that did not have surgery (20.1 months, 95% CI: 10.35, NE) (*p* = 0.86).

**Figure 1:**
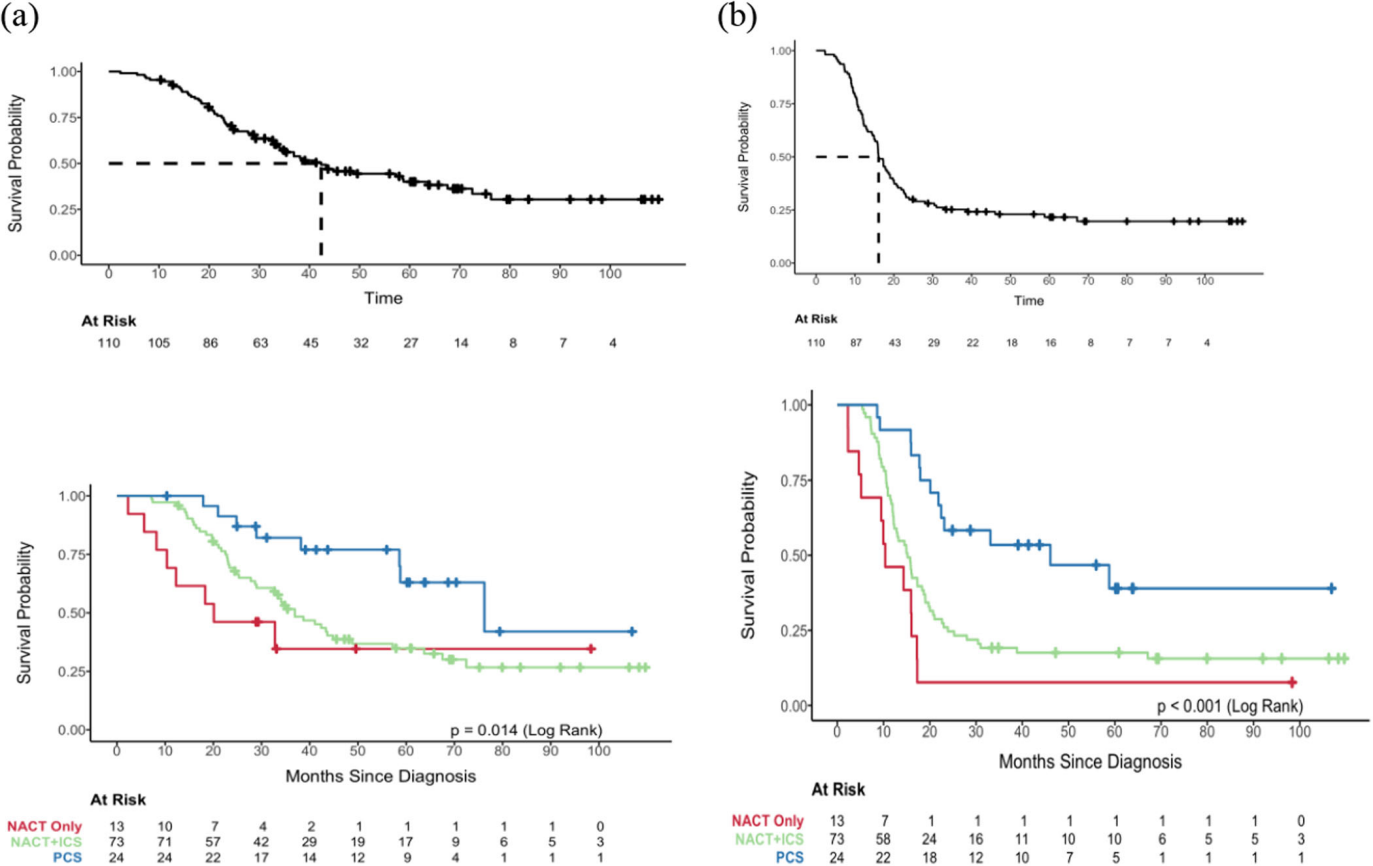
(a) Overall survival and (b) progression‐free survival in women with Stage IVB high‐grade serous ovarian cancer global and by treatment group. ICS, interval cytoreductive surgery; NACT, neoadjuvant chemotherapy; PCS, primary cytoreductive surgery.

The median progression‐free survival overall was 16.1 months (95% CI: 14.9–19.8), differing by treatment group: 46.1 months (95% CI: 22.5–NE) for primary surgery, 15.2 months (95% CI: 12.3–18.7) for interval surgery, and 10.4 months (95% CI: 5.2–NE) for chemotherapy alone (*p* < 0.001) (Figure 1).

### 3.4 Residual Disease and Surgical “Aggressiveness”

Excluding 2 aborted procedures at ICS, and 2 procedures with unreported surgical outcome (both in interval‐surgery group), a total of 93 cytoreductive surgeries had reported outcome. Significantly more patients in the primary‐surgery group compared with interval surgery had upper abdominal procedures (52.4% vs. 16.9%, *p* = 0.001), intrathoracic procedures (33.3% vs. 1.4%, *p* < 0.001), bowel resections (52.4% vs. 8.4%, *p* = 001), and lymph node resection (61.9% vs. 21.1%, *p* < 0.001) (Table 4).

**Table 4:** Cytoreductive surgical procedure characteristics.

Procedure	PCS (*n* = 21)^a^	ICS (*n* = 71)^b^	*p*
Salpingo‐oopherectomy, (unilateral/bilateral), ±total abdominal/radical hysterectomy, omentectomy ± peritonectomy	21 (100%)	71 (100%)	
Lymph node dissection^c^	13 (61.9%)	15 (21.1%)	< 0.001
Pelvic lymph node resection	11 (52.4%)	8 (11.3%)	
Para‐aortic lymph node resection	8 (38.1%)	6 (8.5%)	
Inguinal lymph node resection	3 (14.3%)	2 (2.8%)	
Bowel resection	11 (52.4%)	6 (8.4%)	< 0.001
Small bowel resection	0	3 (4.2%)	
Low anterior resection/Large bowel resection	10 (52.4%)	3 (4.2%)	
Large bowel resection and small bowel resection	1 (4.8%)	0	
Upper abdominal procedures^c^	11 (52.4%)	12 (16.9%)	≈0.001
Diaphragmatic stripping/resection	10 (47.6%)	9 (12.7%)	
Serosal or parenchymal liver resection	7 (33.3%)	4 (5.6%)	
Cholecystectomy/porta hepatis LN resection	3 (14.3%)	0	
Partial gastrectomy/celiac axis LN resection	2 (9.5%)	0	
Splenectomy	6 (28.57%)	5 (7%)	
Distal Pancreatectomy	1 (4.8%)	0	
Intrathoracic procedures	7 (33.3%)	1 (1.4%)	< 0.001
Abdominal wall resection	3 (14.3%)	1 (1.4%)	0.03

^a^ Of the total *n* = 26 who had PCS, 5 were excluded: open and close (*n* = 2) and suboptimal surgical outcome (*n* = 3).

^b^ Of the total cohort of 73, 2 patients planned for an interval cytoreductive surgery had an aborted procedure and excluded from this table.

^c^ The number in these categories represent the total number of patients who underwent one or multiple procedures in the specific category.

Of 24 patients treated with primary surgery, median OS was 76.23 months (95% CI: 58.77, NE) for patients with NGR (*n* = 14), NE (95% CI: 38.16, NE) for those with optimal ≤ 1 cm (*n* = 7), and 28.93 months (95% CI: 20.97, NE) for patients who had suboptimal primary surgery (*n* = 2) (*p* = 0.076).

A total of 20/24 (83.3%) had “aggressive” primary surgery, and 14/24 (58.3%) had NGR surgical outcome. To achieve NGR, 12/14 (85.7%) had “aggressive” surgery, to achieve optimal ≤ 1 cm, 7/7 (100%) had “aggressive” surgery, and 1/3 (33%) suboptimal patient had aggressive surgery (*p* = 0.032). Patients treated with aggressive interval surgery versus nonaggressive primary surgery had similar median OS of 76.23 months (95% CI: 58.6–NE) and 58.77 months (95% CI: 20.87– NE), respectively (*p* = 0.698).

A total of 73 patients underwent interval surgery, 71 of them had reported surgical outcome. Median OS was 41.1 months (95% CI: 29.0–NE) for patients with NGR (*n* = 39), 34.1 months (95% CI: 27.8–NE) for those with optimal ≤ 1 cm (*n* = 26), and 18.5 months (95% CI: 7.4–NE) for patients who had suboptimal/aborted interval surgery (*n* = 6) (*p* = 0.014). In the interval‐surgery group, after excluding 2 patients with aborted procedures, a total of 40/71 (56.3%) had “nonaggressive” interval surgery and 31/71 (43.7%) had “aggressive” interval surgery.

Excluding the 2 patients with unreported surgical outcome, 39/71 (54.9%) had NGR outcome at interval surgery. To achieve NGR, 21/39 (53.9%) patients had nonaggressive and 18/39 (46.2%) had aggressive interval surgery. Optimal cytoreduction < 1 (*n* = 26) was achieved with nonaggressive interval surgery in 14/26 (53.9%) patients and aggressive interval surgery in 12 (46.2%) patients, while suboptimal cytoreduction was the outcome in 5/6 (83.3%) patients with nonaggressive interval surgery and 1/6 (16.7%) patient with aggressive interval surgery (*p* = 0.38) (Table S1).

Patients treated with aggressive interval surgery versus nonaggressive interval surgery had similar median OS of 38.7 months (95% CI: 28.7–67.6) and 35.3 months (95% CI: 23.0–NE), respectively (*p* = 0.67) (Figure S1).

For the 13 patients who were treated with chemotherapy alone, interval surgery was not offered in 6 cases for disease progression, 5 cases for persistent high‐volume disease (with or without decreased functional status), and 2 cases for persistent unresectable disease. Bevacizumab was added to the systemic treatment regimen in 5 of the 13 (38.5%) cases upon the decision not to pursue interval surgery (Table S2).

## 4 Discussion

### 4.1 Summary of Main Results

In this cohort of patients with Stage IVB HGSOC, OS exhibited a hierarchical relationship according to treatment strategy, with primary surgery providing the longest median OS, followed by neoadjuvant chemotherapy and interval surgery, and chemotherapy alone. Disease distribution, particularly supradiaphragmatic lymph node involvement, significantly influenced the choice of CS. More patients in the primary‐surgery group had intrathoracic, upper abdominal, and bowel resections.

Amongst patients undergoing interval surgery, surgical outcome of NGR and optimal cytoreduction was correlated with longer OS compared to suboptimal cytoreduction. To achieve NGR, a similar proportion of patients underwent nonaggressive and aggressive interval surgery, and survival did not differ based on surgical aggressiveness.

### 4.2 Results in the Context of Published Literature

The median OS of women who underwent primary surgery, 76.2 months, is equivalent to the median OS for Stage III disease when compared to historical data [1]. Extensive disease as barrier to debulking surgery in Stage IV epithelial ovarian cancer is also well documented [9]. The median OS and progression‐free survival of the three treatment approaches were similar to that previously described [14, 15]. Though OS and progression‐free survival were expectedly longer compared with studies of mixed histology [16, 17].

Previous studies summarized the survival benefit of achieving NGR surgical outcome in Stage IV patients and highlighted the advantage of optimal cytoreduction (using various residual disease cutoffs) over suboptimal cytoreduction [9, 16, 18, 19, 20]. Additionally, in Stages IIIC–IV patients, survival was similar between those requiring extensive surgery and those not, as long as optimal cytoreduction was achieved [9, 19]. Despite Stage IVB patients being a minority in these studies, our cohort demonstrated similar findings.

Intrathoracic cytoreduction has been shown to be feasible with low morbidity, but its direct survival benefit remains uncertain [19]. Some studies suggest improved progression‐free and OS in carefully selected patients, and recent reviews underscore that extended surgical efforts (including intrathoracic resections) can be beneficial if complete resection is achieved [20]. While our data indicate that patients with supradiaphragmatic disease were less likely to be offered surgery, about one‐third of primary‐surgery patients in our cohort underwent intrathoracic cytoreduction. A prospective evaluation of these practices could clarify selection criteria and further elucidate any survival advantage.

In the literature, multiple parenchymal liver lesions have previously been associated with a decreased likelihood of surgically resectable disease and worse OS and progression‐free survival [9, 16]. A prior analysis comparing women with Stage IVB HGSOC with parenchymal metastases and extra‐abdominal lymph node metastases noted no difference in OS and progression‐free survival between the groups [14].

### 4.3 Strengths and Weaknesses

Our results contribute new evidence on Stage IVB HGSOC prognosis based on treatment strategies, addressing the controversial equipoise between primary surgery and neoadjuvant chemotherapy followed by interval surgery. A small subset (22%) of patients was selected for primary surgery, generally undergoing more extensive surgical procedures yet achieving longer OS. Conversely, those treated with neoadjuvant chemotherapy followed by interval surgery required less‐aggressive procedures to achieve similar rates of NGR disease, but had shorter survival.

This discrepancy in survival outcomes, despite similar surgical results, highlights potential weaknesses related to baseline disease burden, patient factors, and tumor biology. For example, patients' age and performance status may have guided clinicians toward neoadjuvant chemotherapy, and disease distribution could have been more extensive in the interval‐surgery group, limiting the feasibility of primary surgery.

The retrospective design, single‐center nature, and modest sample size are inherent limitations of the study. Variability in patient selection for surgical management prior to 2018 and nonstandardized use of maintenance therapies also limit generalizability. Another key limitation is the lack of objective and standardized tools for reporting surgical outcome.

### 4.4 Implications for Practice and Future Research

Our findings raise the question of whether survival differences between primary‐surgery and interval‐surgery groups primarily reflect underlying disease biology or truly represent the extent of surgical effort. Understanding which patients would benefit most from primary surgery versus neoadjuvant chemotherapy remains an unresolved issue.

Future studies should incorporate more granular assessments of tumor burden, patient factors, and tumor biology, and utilize predictive algorithms to refine patient selection. The development of reliable biomarkers of disease biology will be essential in optimizing clinical decision‐making for Stage IVB HGSOC.

## 5 Conclusions

The prognosis of Stage IVB HGSOC appears to follow a treatment‐based hierarchy: patients undergoing PCS (primary surgery) have the longest survival, followed by ICS (interval surgery), then chemotherapy alone. Although disease distribution strongly influences surgical selection, primary surgery involved more extensive procedures yet was linked to better survival even when surgical outcome was similar to interval surgery. Subgroup analyses by surgical outcome were limited by small sample sizes and wide confidence intervals, and the contribution of underlying disease biology and patient factors influencing the feasibility of primary surgery could not be assessed; these findings should therefore be considered exploratory.

Selecting patients likely to achieve NGR or optimal (≤ 1 cm) cytoreduction remains a crucial determinant of survival in Stage IVB HGSOC, as the minority (17%) of patients who were poor surgical candidates (either had no surgery or a suboptimal surgical outcome at interval surgery) showed the poorest survival.

Future research should focus on the interplay of disease burden, distribution, and biology, and on developing predictive tools to refine patient selection for CS—ultimately improving treatment pathways and outcomes in this challenging population.

## Ethics Statement

This study was approved by the University Health Network Research Ethics Board (CAPCR ID 20‐5945.3).

## Conflicts of Interest

The authors declare no conflicts of interest.

## Synopsis

This retrospective cohort study demonstrates a hierarchical survival pattern in Stage IVB high‐grade serous ovarian cancer: primary cytoreductive surgery achieves the longest survival, followed by interval cytoreductive surgery, and chemotherapy alone. It underscores the importance of achieving a no gross residual surgical outcome, which correlates with improved survival, and highlights the influence of disease distribution on treatment selection.

## Supporting information


**S2. Supplementary Figure 1:** Overall survival of women with stage IVB high grade serous ovarian cancer treated with cytoreductive surgery by non‐aggressive vs aggressive surgery*. **S1. Supplementary Table 1:** Gross residual tumor size after non‐aggressive vs aggressive cytoreductive surgery*. **S3. Supplementary Table 2:** Summary of non‐surgical patients with stage IVB high grade serous ovarian cancer.

## Data Availability

The data that support the findings of this study are available on request from the corresponding author. The data are not publicly available due to privacy or ethical restrictions.
