# Supplementary material for: Treatment Strategy and Residual Disease as Determinants of Survival in Stage IVB High‐Grade Serous Ovarian Cancer: A Retrospective Cohort Study
Source: J Surg Oncol. 2025 Nov 24;133(1):73–80. doi: 10.1002/jso.70142 (PMC12747705; doi:10.1002/jso.70142)
Supplement: Supplementary file 1 — S2. Supplementary Figure 1: Overall survival of women with stage IVB high grade serous ovarian cancer treated with cytoreductive surgery by non‐aggressive vs aggressive surgery*. S1. Supplementary Table 1: Gross residual tumor size after non‐aggressive vs aggressive cytoreductive surgery*. S3. Supplementary Table 2: Summary of non‐surgical patients with stage IVB high grade serous ovarian cancer. [file JSO-133-73-s001.docx]

**Supplementary.**

**S1. Supplementary Table 1.** Gross residual tumor size after non-aggressive vs aggressive cytoreductive surgery*

|  | **Non-aggressive PCS (n=4)** | **Aggressive PCS (n=20)** | ***Total PCS (n=24)*** | P value |
| --- | --- | --- | --- | --- |
| **Residual tumor after PCS** |  |  |  | **0.032** |
| *No gross residual tumor* | *2/14 (14.3%)* | *12/14 (85.7%)* | *14(58.3%)* |  |
| *Optimal cytoreduction*  *(*$\leq$*1cm gross residual tumor)* | *0/7 (0%)* | *7/7 (100%)* | *7 (29.1%)* |  |
| Suboptimal cytoreduction  (>1 cm gross residual tumor)  /Aborted procedures^§^ | 2/3 (66.7%) | 1/3 (33.3%) | 3(12.5%) |  |
|  | **Non-aggressive ICS (n=40)^*^** | **Aggressive ICS (n=31)^*^** | ***Total ICS (n=71)^^^*** | P value |
| **Residual tumor after ICS** |  |  |  | **0.38** |
| *No gross residual tumor* | *21/39 (53.9%)* | *18/39 (46.2%)* | *39(54.9%)* |  |
| *Optimal cytoreduction*  *(*$\leq$*1cm gross residual tumor)* | *14/26 (53.9%)* | *12 (46.2%)* | *26(36.6%)* |  |
| Suboptimal cytoreduction  (>1 cm gross residual tumor)  /Aborted procedures^§^ | 5/6 (83.3%) | 1/6 (16.7%) | 6 (8.5%) |  |

Abbreviations: ICS– interval cytoreductive surgery

^ for 2 patients the surgical outcome was not reported, those were not included in this table

*Non-aggressive surgery was defined as including total abdominal hysterectomy, bilateral salpingo-oopherectomy, omentectomy, peritonectomy and pelvic lymph node dissection

Aggressive surgery additionally included resection of one or more of the following: diaphragm, liver, spleen, thorax, bowel, para-aortic lymph nodes, inguinal lymph nodes or abdominal wall.

**S2. Supplementary Figure 1:** Overall survival of women with stage IVB high grade serous ovarian cancer treated with cytoreductive surgery by non-aggressive vs aggressive surgery*


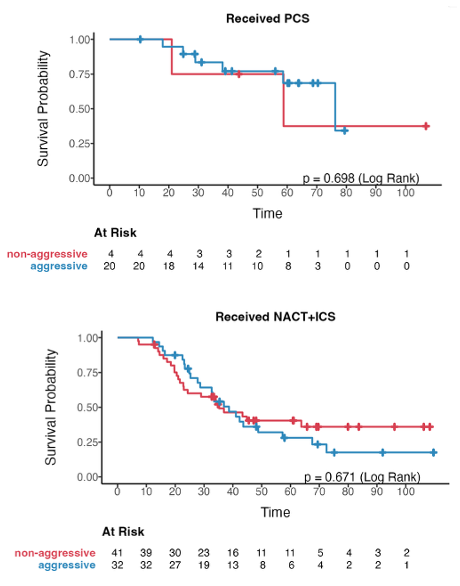


Abbreviations: PCS- primary cytoreductive surgery, NACT – neoadjuvant chemotherapy, ICS– interval cytoreductive surgery

*Non-aggressive surgery was defined as including total abdominal hysterectomy, bilateral salpingo-oopherectomy, omentectomy, peritonectomy and pelvic lymph node dissection

Aggressive surgery additionally included resection of one or more of the following: diaphragm, liver, spleen, thorax, bowel, para-aortic lymph nodes, inguinal lymph nodes or abdominal wall.

**S3. Supplementary Table 2:** Summary of non-surgical patients with stage IVB high grade serous ovarian cancer

|  | Age | Ca125 | | Bevacizumab | Reason(s) ICS was not offered | | | | |
| --- | --- | --- | --- | --- | --- | --- | --- | --- | --- |
|  |  | Baseline | After NACT |  | Age | ECOG ≥2 | Persistent unresectable disease | Persistent high-volume disease | Disease progression on CT |
| 1 | 46 | 131 |  |  |  | 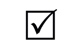 |  |  | 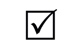 |
| 2 | 73 | 24 375 | 1 832 |  |  |  |  |  | 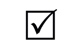 |
| 3 | 82 | 1 279 | 496 |  |  | 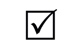 |  | 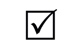 |  |
| 4 | 43 | 451 | 451 |  |  | 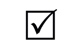 |  | 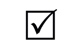 |  |
| 5 | 58 | 5 385 | 1 984 | 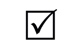 |  |  |  |  | 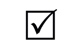 |
| 6 | 62 | 1 317 | 12 | 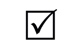 |  |  |  |  | 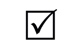 |
| 7 | 80 |  | 4 |  | 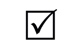 |  |  | 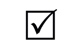 |  |
| 8 | 50 | 727 | 71 | 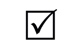 |  |  |  | 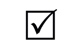 |  |
| 9 | 77 | 12 582 | 144 | 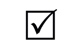 |  |  | 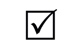 |  |  |
| 10 | 67 | 1 373 | 37 |  |  |  |  | 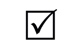 |  |
| 11 | 73 | 3 417 | 960 |  |  |  |  |  | 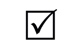 |
| 12 | 58 | 34 | 13 |  |  |  | 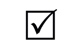 |  | 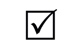 |
| 13 | 50 | 1 595 | 106 | 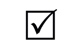 |  |  | 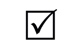 |  |  |

Abbreviations: ICS– interval cytoreductive surgery, NACT – neoadjuvant chemotherapy, ECOG – Eastern Cooperative Oncology Group, CT – Computer tomography
